# Supplementary material for: Clustering Analysis Identified Three Long COVID Phenotypes and Their Association with General Health Status and Working Ability
Source: J Clin Med. 2023 May 23;12(11):3617. doi: 10.3390/jcm12113617 (PMC10253616; doi:10.3390/jcm12113617)
Supplement: Supplementary file 1 [file jcm-12-03617-s001.zip › jcm-2396546-supplementary (renumber).pdf]

Supplementary Figure S1: Chart flow and steps of data analysis.

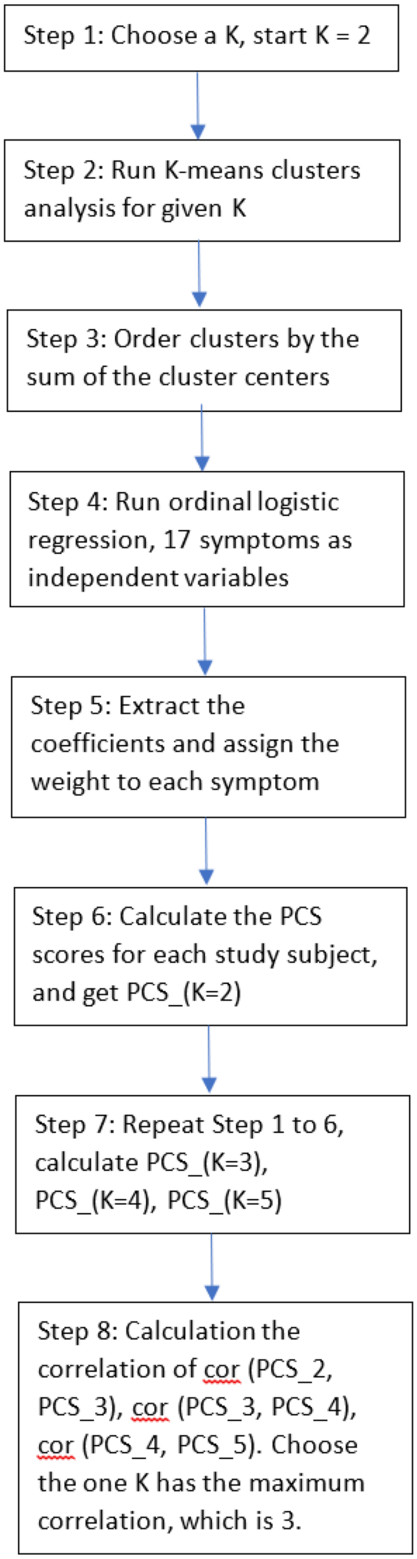

Supplementary Figure S2: The boxplot of the PCS scores for three K-Means clusters (n = 506). The boxes show the median, and 25% and 75% quantiles. The whiskers show the most extreme data points or 1.5 times of the interquartile rang (IQR) from the box. Cluster I (n = 299) has the minimum sum of the cluster center, Cluster III (n = 87) has the maximum sum of the cluster center, the sum of the cluster center of Cluster II (n = 120) is in the middle.

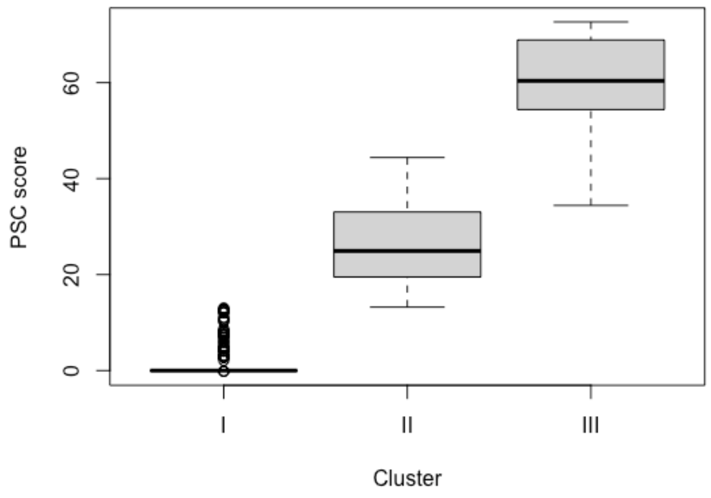

Supplementary Figure S3: The distribution of the post-COVID syndrome (PCS) score in the 3 cohorts (non-hospitalized, hospitalized, post-COVID).

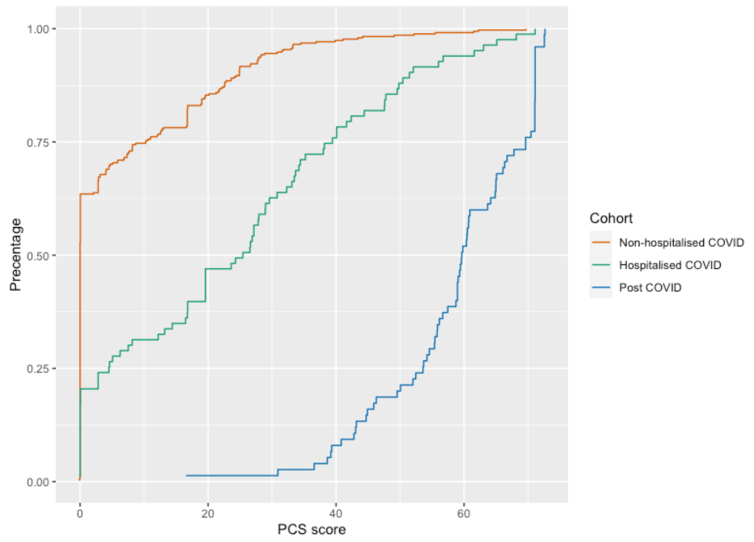

Supplementary Figure S4: Principal component analysis were performed on the 17 symptom complex indicators that form the PCS score definition in all the three cohorts together (n = 506). The first two principal components, which account for 55.4% (horizontal axis) and 6.2% (vertical axis) of the variance of the indicators, are displayed. Individuals are colored according to their respective cluster affiliations (I, II, III). The K-Means centers for each cluster are also shown. Cluster I (n = 299) has the minimum sum of the cluster center, Cluster III (n = 87) has the maximum sum of the cluster center, the sum of the cluster center of Cluster II (n = 120) is in the middle.

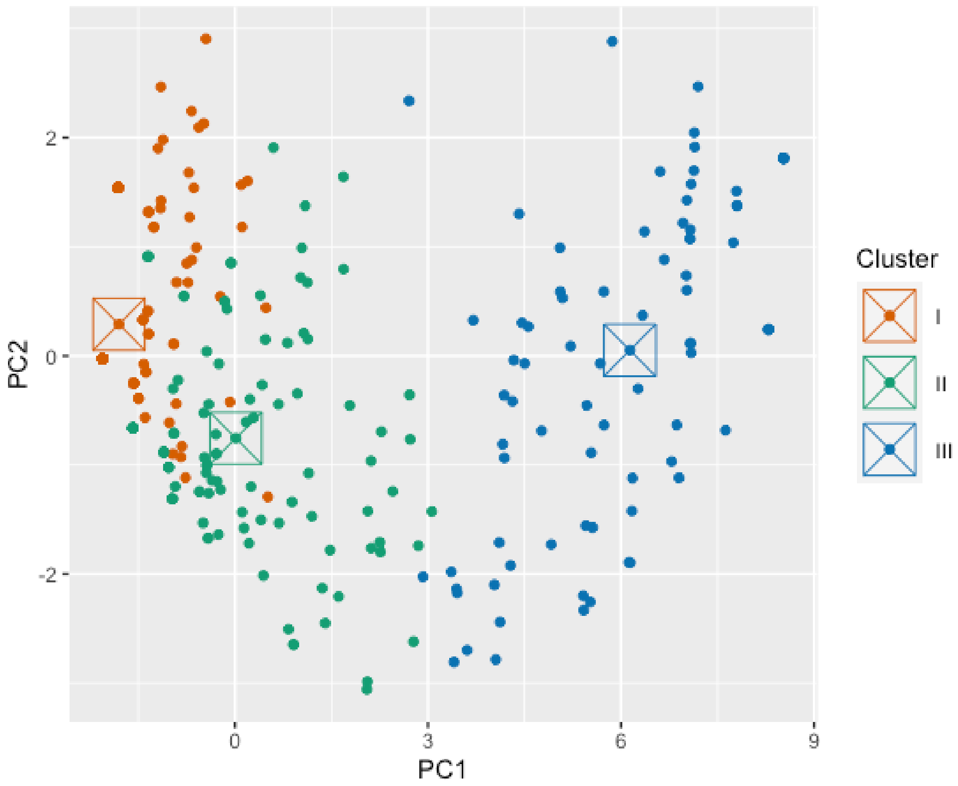

Supplementary Table S1: The proportion of the missing values for each variable for all three cohorts combined (n = 584).

**Variable**	**% missing values (n)**
GI Symptom	11.47 (67)
Skin problems	11.30 (66)
Sore throat	11.30 (66)
Cough	10.96 (64)
Nasal Symptom	10.62 (62)
Chest pain	10.62 (62)
Heart palpitation	10.62 (62)
Vertigo	10.45 (61)
Anxiety mood	10.45 (61)
Muscle and joints pain	10.27 (60)
Dyspnea	9.93 (59)
Depressive mood	9.93 (59)
Impaired taste and smell	9.76 (57)
Headache	9.42 (55)
Memory and concentration problems	9.42 (55)
Sleep problems	9.25 (54)
Fatigue	8.39 (49)

Supplementary Table S2: The Pearson correlation between Post COVID Scores (PCS) for different numbers of clusters based on regression on all three sub-cohorts (n = 506).

**Pearson correlation**	**All three sub-cohorts together**
2 and 3 clusters	0.980
3 and 4 clusters	0.992
4 and 5 clusters	0.989

Supplementary Figure S5: Principal component analysis were performed on the 17 symptom complex indicators that form the PCS score definition in the train data set (n = 407). The first two principal components, which account for 55.4% (horizontal axis) and 6.4% (vertical axis) of the variance of the indicators, are displayed. Individuals are were colored according to their respective cluster affiliations (I, II, III). The K-Means centers for each cluster are also shown. Cluster I (n = 234) has the minimum sum of the cluster center, Cluster III (n = 69) has the maximum sum of the cluster center, the sum of the cluster center of Cluster II (n = 104) is in the middle.

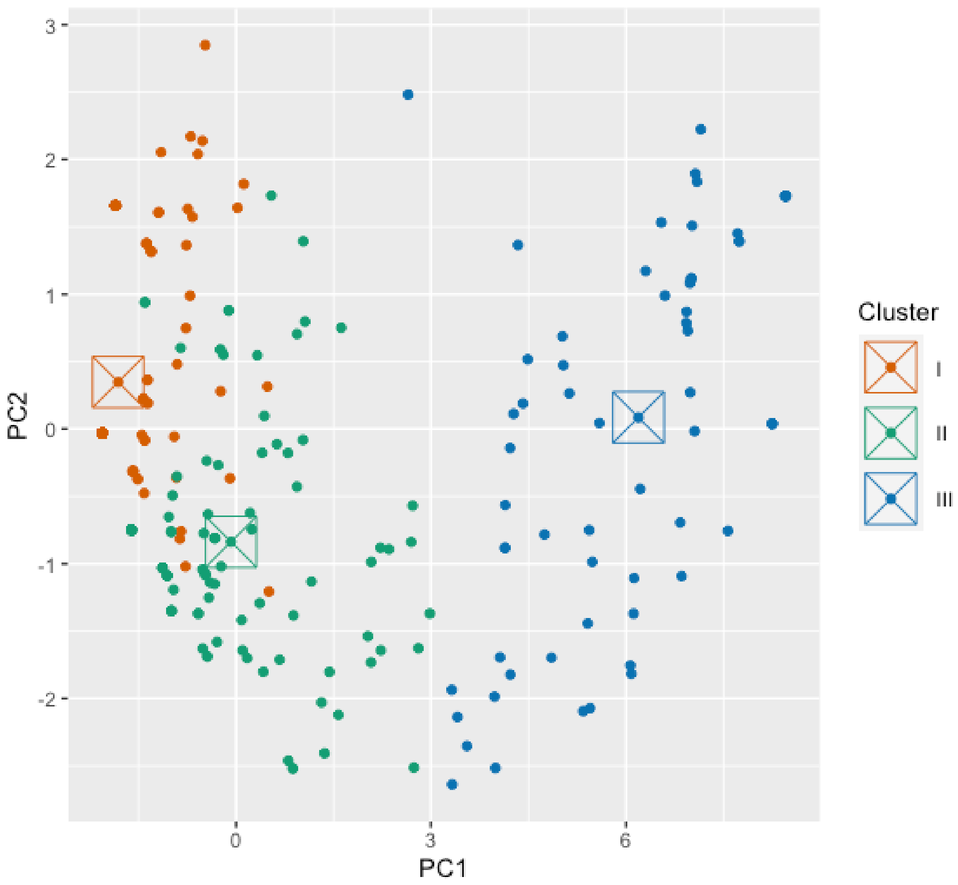

.

Supplementary Figure S6: The boxplot of the PCS scores for three K-Means clusters in the train data set (n = 407). The boxes show the median, and 25% and 75% quantiles. The whiskers show the most extreme data points or 1.5 times of the interquartile rang (IQR) from the box.
Cluster I (n = 234) has the minimum sum of the cluster center, Cluster III (n = 69) has the maximum sum of the cluster center, the sum of the cluster center of Cluster II (n = 104) is in the middle.

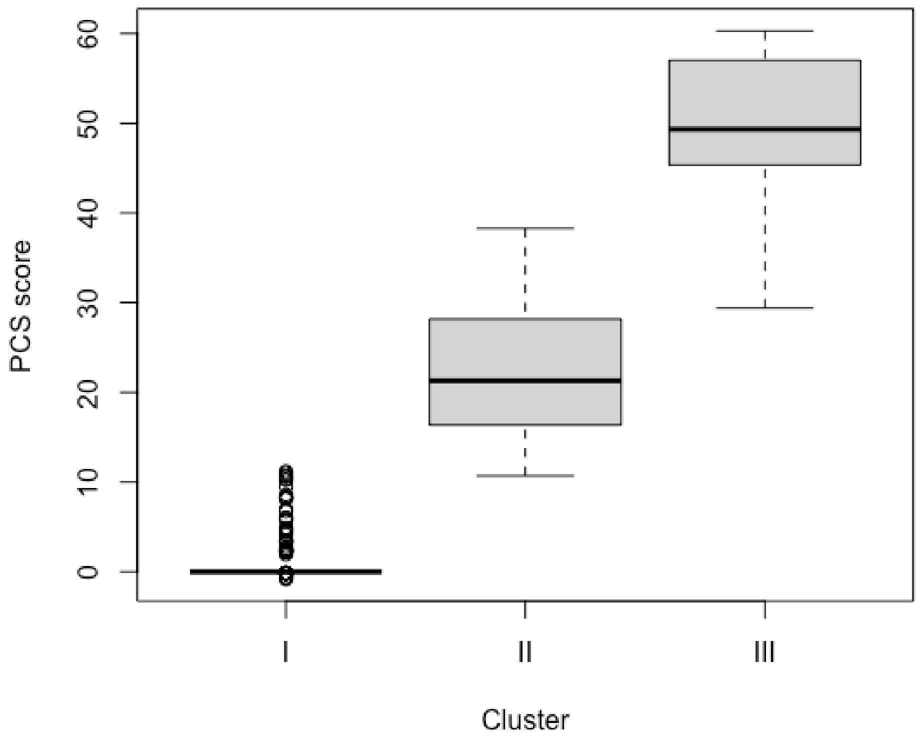

Supplementary Figure S7: The frequency of long-term remining symptoms in three cohorts in the train set (n = 407). Non/mile (PCS ≤ 12, n = 235), Moderate (PCS > 12 and PCS ≤ 40, n = 106) and Severe (PCS > 40, n = 66).

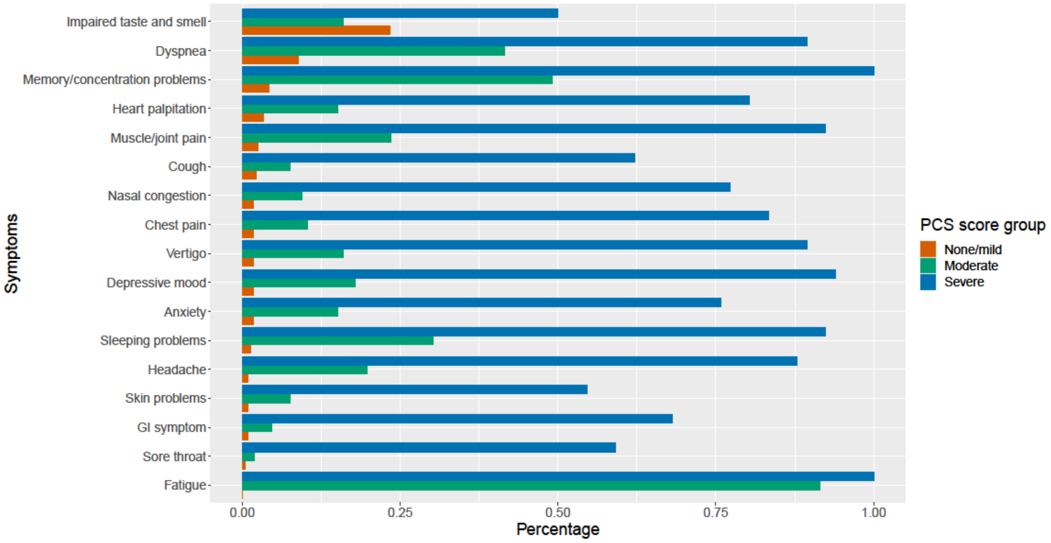

Supplementary Table S3: The Pearson correlation between Post COVID Scores (PCS) for different numbers of clusters based on regression on train-data (used PCS score weight from train data on both train and test data).

**Pearson correlation**	**Train-data (n = 407)**	**Test-data (n = 99)**
2 and 3 clusters	0.947	0.955
3 and 4 clusters	0.985	0.988
4 and 5 clusters	0.986	0.986

Supplementary Table S4. Post COVID score (PSC) development by doing K-Means clustering with 3 clusters and ordinal logistic regression, on train-data (n = 407). Cluster I (n = 234) has the minimum sum of the cluster center, Cluster III (n = 69) has the maximum sum of the cluster center, the sum of the cluster center of Cluster II (n = 104) is in the middle.

**No**	**Symptom complex**	**Train cluster center I (n = 234)**	**Train cluster center II (n = 104)**	**Train cluster center III (n = 69)**	**Regression coefficient**	**PCS* score weight**
2	Fatigue	0.000	0.913	0.986	14.465	14.5
15	Memory and concentration problems	0.0395	0.481	1.000	8.337	8.3
4	Sore throat	0.004	0.019	0.565	4.827	4.8
3	Muscles and joints pain	0.026	0.221	0.913	4.556	4.6
1	Cough	0.021	0.067	0.609	3.678	3.7
6	Headache	0.009	0.183	0.870	3.635	3.6
7	Vertigo	0.017	0.144	0.884	3.489	3.5
10	Chest pain	0.017	0.087	0.826	3.314	3.3
12	GI symptoms	0.009	0.009	0.667	3.034	3.0
11	Heart palpitation	0.034	0.135	0.797	2.541	2.5
5	Dyspnea	0.085	0.413	0.884	2.372	2.4
14	Depressive mood	0.017	0.173	0.913	2.222	2.2
13	Anxiety mood	0.017	0.135	0.754	1.919	1.9
17	Sleep problems	0.013	0.288	0.913	1.914	1.9
16	Impaired taste and smell	0.235	0.154	0.493	-0.157	-0.2
9	Nasal symptoms	0.017	0.087	0.754	-0.664	-0.7
8	Skin problems	0.009	0.067	0.536	-2.462	-2.5

*Post COVID syndrome (PCS) score.

Supplementary Table S5: The characteristic of the test clusters based on PCS score in test-data. (n = 99)

**Characteristics**	**None/mild PCS* score ≤ 12 (n = 64)**	**Moderate PCS* score > 12 and ≤ 40 (n = 20)**	**Severe PCS* score > 40 (n = 15)**	**P values,[+]**	**P values adjusted,[+]**
Sociodemographic and lifestyle					
Age (mean, sd)	43.9 (13.5)	51.9 (11.2)	51.2 (12.7)	**0.023**	**0.041**
Female (n, %)	45 (70.3)	13 (65.0)	11 (73.3)	0.855	0.855
Country of birth (Sweden or not) (n, %)	52 (81.2)	14 (70.0)	14 (93.3)	0.220	0.296
Education level (n, %)				0.733	0.824
Up to Gymnasium	23 (35.9)	7 (35.0)	7 (46.7)		
Two years	5 (7.8)	3 (15.0)	2 (13.3)		
Three years	36 (56.3)	10 (50.0)	6 (40.0)		
Working status (n, %)				**0.001**	**0.002**
Working	58 (92.0)	18 (94.7)	8 (53.4)		
Parental leave	1 (1.6)	0 (0.0)	0 (0)		
Looking for a job	1 (1.6)	1 (5.3)	0 (0)		
Retired	2 (3.2)	0 (0.0)	2 (13.3)		
Sick leave	1 (1.6)	0 (0.0)	5 (33.3)		
Student	0 (0)	0 (0.0)	0 (0)		
Marital status (n, %)				0.230	0.296
Married	21 (32.8)	10 (50.0)	5 (33.3)		
Sambo	26 (40.6)	3 (15.0)	7 (46.7)		
Divorced	10 (15.7)	4 (20.0)	0 (0.0)		
Widower	3 (4.7)	1 (5.0)	0 (0.0)		
Single	4 (6.2)	2 (10.0)	3 (20.0)		
Smoking (n, %)				**< 0.001**	**<0.001**
Never Smoked	51 (81.0)	12 (63.2)	0 (0)		
Ex-smoker	10 (15.9)	3 (15.8)	7 (46.7)		
Current smoker	2 (3.1)	4 (21.0)	8 (53.3)		
Snus (n,%)	50 (79.4)	8 (44.4)	3 (20.0)	**< 0.001**	**<0.001**
Pre-existing comorbidities (n, %) and BMI					
BMI (mean, sd)	25.2 (4.2)	27.0 (4.4)	30.2 (6.8)	**0.002**	**0.004**
Hypertension	8 (12.5)	3 (15.0)	4 (26.7)	0.387	0.532
Heart disease	2 (3.1)	2 (10.0)	1 (6.7)	0.450	0.550
Hypo/hyperthyroidism	5 (7.8)	2 (10.0)	1 (6.7)	0.930	0.930
Diabetes	0 (0)	3 (15.0)	0 (0)	**0.002**	**0.005**
Lung disease	6 (9.4)	6 (30.0)	9 (60.0)	**<0.001**	**<0.001**
Liver disease	0 (0)	0 (0)	0 (0)	NA	NA
Cancer	2 (3.1)	1 (5.0)	2 (13.3)	0.267	0.420

Immunosuppressive treatment	3 (4.7)	2 (10.0)	1 (25.0)	0.682	0.750
Depression/Anxiety	16 (25.0)	8 (40.0)	9 (60.0)	**0.027**	0.050
Chronic pain	3 (4.7)	2 (10.0)	6 (40.0)	**<0.001**	**0.002**
Other measurements					
Symptom severity at onset, median (IQR)	2 (2, 3)	4 (3, 4)	4 (3.5, 4.5)	**<0.001**	**<0.001**
Hospitalised, n (%)	6 (9.4)	10 (50.0)	6 (40.0)	**0.001**	**0.002**
COVID infection laboratory confirmed, n (%)	64 (100)	19 (95)	12 (80)	**0.003**	**0.004**
Number of months from infection onset, mean (SD)	12.0 (0)	13.1 (3.6)	20.4 (6.5)	**<0.001**	**<0.001**
Mean number of remaining symptoms, mean (SD)	0.5 (0.9)	5.2 (2.9)	13.3 (2.2)	**< 0.001**	**<0.001**
Health before COVID, median (IQR)	90 (85, 95)	95 (91.3, 98.8)	95 (70, 95)	0.579	0.579
Health today, median (IQR)	85 (80, 95)	70 (54, 80)	45 (20, 60)	**<0.001**	**<0.001**
Difference health now and before CVOID, median (IQR)	0 (10, 0)	-20 (-30, -11)	-25 (-33, -13)	**0.023**	**0.029**
Workability before COVID, median (IQR)	10 (9, 10)	10 (9, 10)	10 (8.5, 10)	0.240	0.264
Workability now, median (IQR)	9 (8, 10)	7 (6, 8)	4 (1, 6)	**< 0.001**	**< 0.001**
Difference working ability now and before COVID, median (IQR)	0 (0, 0)	-2 (-3, -2)	-6 (-8, -3)	**< 0.001**	**< 0.001**

*Post COVID syndrome (PCS) score; +, P values shown in bold are significant under level 0.05.
